# The Neurophysiological and Psychological Mechanisms of Qigong as a Treatment for Depression: A Systematic Review and Meta-Analysis

**DOI:** 10.3389/fpsyt.2019.00820

**Published:** 2019-11-18

**Authors:** Wendy Wing Yan So, Shuhe Cai, Suk Yu Yau, Hector Wing Hong Tsang

**Affiliations:** ^1^Department of Rehabilitation Sciences, Hong Kong Polytechnic University, Hong Kong, Hong Kong; ^2^Department of Orthopaedic Rehabilitation, Affiliated Rehabilitation Hospital of Fujian University of Traditional Chinese Medicine, Fuzhou, China

**Keywords:** qigong, complementary and alternative medicine, neurophysiological mechanism, depression, anti-depressive

## Abstract

**Objective:** An increasing number of studies have shown the anti-depressive effect of qigong. However, its underlying mechanism remains poorly understood. This study aims to systematically review and meta-analyze existing literature on the mechanism of qigong in reducing depression.

**Method:** The review process followed the Preferred Reporting Items for Systematic Reviews and Meta-Analyses (PRISMA) guidelines. Randomized controlled trials of qigong were searched from PsycINFO, PubMed, Embase, ScienceDirect, and Academic Search Premier from inception to December 2018. Studies which involved depression and any neurophysiological or psychological mechanisms as outcomes were included. Publication bias was tested before conducting meta-analysis. Two independent raters were involved for the entire review process.

**Results:** A total of nine studies were identified which covered both neurophysiological and psychological mechanisms. Among these selected studies, seven were involved in meta-analysis, which suggested that qigong was effective in alleviating depression (standardized mean difference, SMD = −0.27, *p* < 0.05, *I* ^2^ = 27%). A significant effect was also found for diastolic blood pressure (SMD = −1.64, *p* < 0.05, *I* ^2^ = 31%). However, no significant effect was found for cortisol level and systolic blood pressure.

**Conclusions:** This review shows that qigong is effective in reducing depression through activating the parasympathetic nervous system. Future studies with higher quality of research methodology with less selection and attrition bias should be conducted to unravel the possible anti-depressive effect of qigong.

## Introduction

Depression is a common and serious mental health disorder that is estimated to affect 350 million people worldwide (1). The most common type of depression is major depressive disorder characterized by depressed mood, loss of interest or pleasure, and altered cognition and which is expected to become the second leading contributor to overall disease burden by 2030 (2). The large number of people suffering from depression has caught researchers’ attention. In order to seek effective treatment for depression, understanding of the pathophysiological changes in patients with depression is an urgent need. Change in the endocrine and immune systems in patients with depression is one of the current major research foci on depression.

The limbic system in the human brain is responsible for emotion regulation. Depressive symptoms are likely to be related to dysfunction in the brain networks linking the limbic system and cortical regions (3). More importantly, the limbic system is responsible for controlling the function of the hypothalamic–pituitary–adrenal (HPA) axis. Under stressful situations, the HPA axis will be activated. Once the HPA axis is hyperactive, an increased amount of glucocorticoid will be released into the body, which is thought to be an etiological factor of depression (4). This constitutes a pathophysiological explanation why an increased level of cortisol is often observed in patients with depression. In addition, desensitization of the glucocorticoid receptor (GR) may play a role in the pathology of depression. GR is extensively distributed throughout the hippocampus and is responsible for the feedback mechanism that regulates the HPA axis (5). When GR in the hippocampus detects an increased level of cortisol, the hippocampus regulates the hypothalamus to decrease corticotrophin-releasing hormone and thus reduce the level of cortisol *via* a negative feedback loop. However, patients with depression have impaired GR and are not able to control this process (6).

Considering that the HPA axis is linked to immune response, abnormal activities in the immune system are found to be concurrent with depression (7). An overactivation of the innate immune system has been shown in individuals with depression (8). Notably, there is an association between pro-inflammatory cytokine alterations and depression (9). Several studies have reported that glucocorticoids will increase anti-inflammatory cytokine levels and decrease pro-inflammatory cytokine levels (10). Pro-inflammatory cytokines may produce many neurological changes that are related to the pathology of depression, including decreased neurogenesis, regional brain abnormalities, changes in the monoamine system, and neurodegeneration (11). Based on the above literature, current research suggests that both the endocrine and immune systems may play a role in depression.

Currently, therapeutic administration of antidepressant medication is the most common treatment for depression. Antidepressants have anti-inflammatory properties affecting the levels of pro-inflammatory cytokines. Serotonin reuptake inhibitors (SSRIs), a common type of antidepressant, mainly function in raising the levels of serotonin and reducing cortisol secretion in the brain (12). Also, SSRIs are able to increase the concentration of anti-inflammatory cytokines in the serum of depressed patients (13). Although taking SSRIs could be effective in reducing depressive symptoms, there are many side effects found in depressed patients following drug treatment (14). Side effects of currently available drug treatment include headache, sedation, sleep disturbance, alteration of cardiovascular function, and bone loss (12). As a result, researchers are seeking better options for treating depression.

Recently, a growing number of studies are investigating the effect of qigong on depression (15). Qigong is an ancient Chinese healing art having a history of 7,000 years (16). According to the Traditional Chinese Medicine (TCM) theory, it is a mind–body discipline which does not only focus on the health-related benefits of physical fitness but is integrated with mindfulness-based practices as well as somatic experiences to improve mental health (17). The basic components of qigong involve concentration, relaxation, meditation, breathing regulation, body posture, and movement (18). Qigong is often performed in association with abdominal breathing and some mindfulness elements. By combining movement and concentration, individuals experience the enhanced flow of “qi,” which is considered as the vital life-force energy within the body (17). In TCM theories, qigong is a generic term to refer to all kinds of mind–body practices with a view to mobilize the flow of qi inside the human body. Smooth flow of qi along meridian channels in the body is considered a condition of health (19). Due to the long history of qigong, many different types and forms are now in place. The most popular protocols include Wuqinxi, Baduanjin, Yi Jin Jing, Liu Zi Jue, and Ma Wang Dui Dao Yin Shu (20). In China, approximately 5% of the 1.3 billion people practice qigong to improve their health and prevent diseases (21). Moreover, qigong has become more and more popular in foreign countries. In recent years, people from Western countries have also started to practice qigong. According to Lauche et al. (22), 7.38 million US adults are practicing Tai chi or qigong on a daily basis.

Practicing qigong can be very beneficial to our physical and mental health (23). A recent systematic review shows that qigong practice can improve quality of life, sleep quality, balance, handgrip strength, trunk flexibility, blood pressure, and heart rate (24). Another review puts focus on the psychological benefits of qigong and suggests that it can reduce stress and anxiety in healthy adults (25). Due to the wide range of physical and mental health benefits, qigong is well placed to assist in improving the health of many people.

It is noteworthy to know that qigong is not only practiced by healthy people but also patients in various clinical settings. Healthcare professionals have been applying it as an alternative treatment among patients with diversified clinical conditions (26, 21, 27). Recently, a systematic review found that qigong was effective in reducing depressive symptoms (28). Most importantly, it was found that qigong exercise produced a similar effect to SSRIs (4) on depressive symptoms (29). If qigong is confirmed to be an evidence-based adjunct intervention, it is likely that the dosage of medication and, hence, its side effects could be reduced.

Although more and more evidence bolsters that qigong is useful in treating depression, its underlying mechanism remains a mystery. Some researchers therefore began to unravel the mechanism of the anti-depressive effects of qigong. Tsang and Fung (14) performed literature review and proposed possible psychological and neurobiological mechanisms that may explain the anti-depressive effect of qigong. Tsang and Fung suggested that qigong is able to reduce stressful signals from the limbic system and thus lower the level of HPA activity. Although research that investigates the psychological mechanism of qigong on depression is sparse, Tsang and Fung suggested that self-efficacy is an important psychological mechanism that regulates depressive symptoms through the use of qigong.

Unfortunately, most of the studies in this area only focus on one aspect of the mechanism. Most reviews did not provide a full picture of how qigong may affect our physical and mental health. For example, Lee et al. (30) investigated the effect of qigong on the neuroendocrine system, Tsang et al. (12) investigated the psychological mechanism of qigong practice, and Li et al. (31) investigated the effect of qigong on the physiological system. A surge in the practice of qigong in recent years has led to an increased need in understanding the underlying biological mechanisms leading to improved physical and mental health. The objective of the present systematic review and meta-analysis is to identify possible neurophysiological and psychological symptom domains of the anti-depressive effects of qigong. To our knowledge, this is the first study that used a meta-analytic approach to address the above issue.

## Methods

The review procedure follows the Preferred Reporting Items for Systematic Reviews and Meta-Analyses (PRISMA) guidelines (32).

### Data Source

Studies were searched from multiple databases, including PsycINFO, PubMed, Embase, ScienceDirect, and Academic Search Premier from inception to December 2018. The same set of search terms was used to search for all of the databases mentioned above. Since a large number of search terms were used in this review, the search terms and strategies used are presented in Appendix 1. Reference lists of relevant studies were screened.

### Study Selection

All results’ title and abstract were exported to EndNote X7 for removing duplications and further screening of eligibility. In order to assess the eligibility of each study, a set of inclusion and exclusion criteria was established. Studies were included if: 1) the independent variable was qigong; 2) the dependent variable involved any types of neurophysiological or psychological indicators and a measurement of depression; 3) they were randomized controlled trials or quasi-experimental studies; 4) they were published in English; and 5) they were full-length articles in peer-reviewed journals. Exclusion criteria included studies that 1) were qualitative in nature; 2) only described the research protocols; 3) use external qigong as independent variable; 4) had no assessment on depression; 5) use a depressed sample with comorbid psychiatric conditions; 6) no underlying biological mechanism was investigated; and 7) were written in languages other than English. All of the studies were screened by two independent reviewers. Disagreement was resolved through discussion until a compromise was reached.

### Data Extraction

The following data were extracted from the studies: 1) sample size; 2) study population; 3) types and duration of intervention; 4) control group information; 5) types of outcomes; and 6) study results according to PRISMA guidelines. This process was performed by two independent reviewers to ensure the accuracy of the information.

### Quality Assessment

The risk of bias tool developed by Cochrane Collaboration was used to assess all the included studies. Eight categories of bias, including selection bias, performance bias, detection bias, attrition bias, reporting bias, and other bias which did not fit into any of the above categories, were assessed. Two independent reviewers rated each item for all the included studies. Their results were compared, and disagreement was resolved through discussion until a consensus was reached.

### Data Synthesis and Meta-Analysis

Studies which included a comparison group were meta-analyzed. But a minimum of three studies with the same outcome were needed. Since different depression scales were used in different studies, the standardized mean difference (SMD) was adopted for the meta-analysis of the effect of qigong on depression. For other outcomes, mean differences were used. Publication bias was tested using Egger’s regression. If the *p* value was less than 0.1 in the two-tailed test, the outcome was not included in the meta-analysis. If the *p* value was larger than 0.1, meta-analysis was conducted. Review Manager 5.3, developed by the Cochrane Collaboration (2014), was used to analyze the results of studies included in the meta-analysis. Heterogeneity was tested and *I* ^2^ was reported. A random effects model was used for heterogeneity (*p* < 0.05), while a fixed effects model was used when heterogeneity was not significant (*p* > 0.05) (33).

## Results

### Search Results

**Figure 1:**
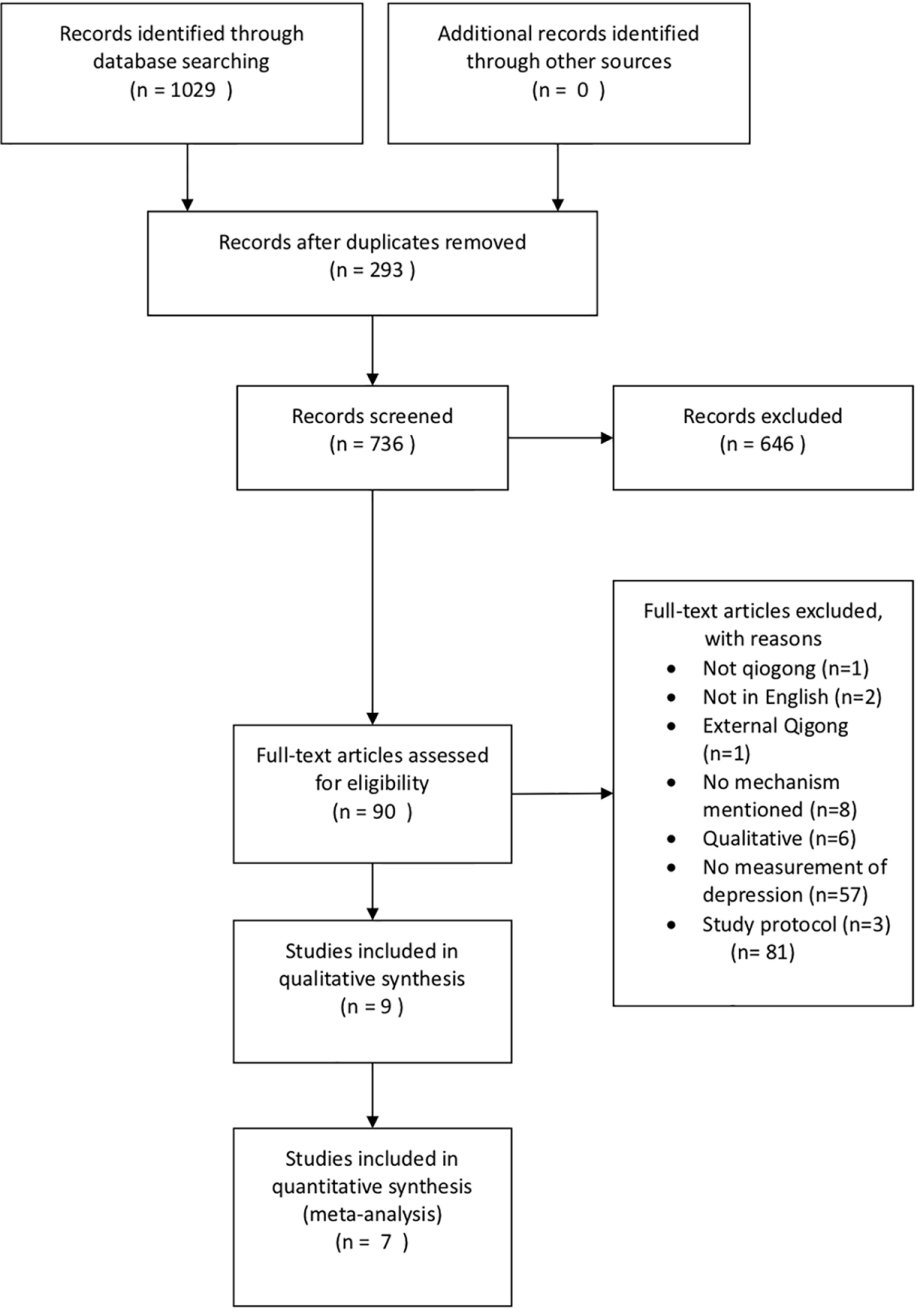
PRISMA flow diagram. Seven studies were included in the meta-analysis for depression and four studies were included in the meta-analysis for blood pressure.

A total of 1,029 articles were identified from the databases based on our proposed keywords. After initial screening of the titles and abstracts, both reviewers identified 90 articles that were potentially suitable to be included in this review. During full-text screening, we found 57 studies without any measurement of depression, eight studies without mentioning any mechanism, six studies adopting qualitative methods, three studies which were protocols, two studies not in English, one study which did not use qigong as the intervention, and one study which used external qigong as intervention. Hence, these studies were excluded. After assessing full-text articles, nine studies were eligible following our selection criteria listed earlier (**Figure 1**).

### Description of the Included Studies

Among the included studies, the sample size varied from 24 to 116 participants. Participants were either adults or older adults, ranging from 18 to 84 years of age. Regarding the target population, five studies used healthy subjects, two studies used patients with depression, one study used patients with breast cancer, and one used adults with hypertension. Only one study used patients with major depressive disorder as subjects, and they were on escitalopram. The types of qigong intervention varied across the included studies. Eight Section Brocades (or Baduanjin) was used in two studies, whereas the remaining seven studies used seven different interventions, including Yi Jin Ten-Section Brocades, Tai chi, Chan Ming Gong, self-healing qigong, Guolin New Qigong, Guolin Qigong, and Laughing Qigong. For the control group, four studies used waitlist control, two studies used newspaper reading program, one study used health education program, one used conventional exercise, and one used treatment as usual.

#### Effect of Qigong on Depression

**Figure 2:**
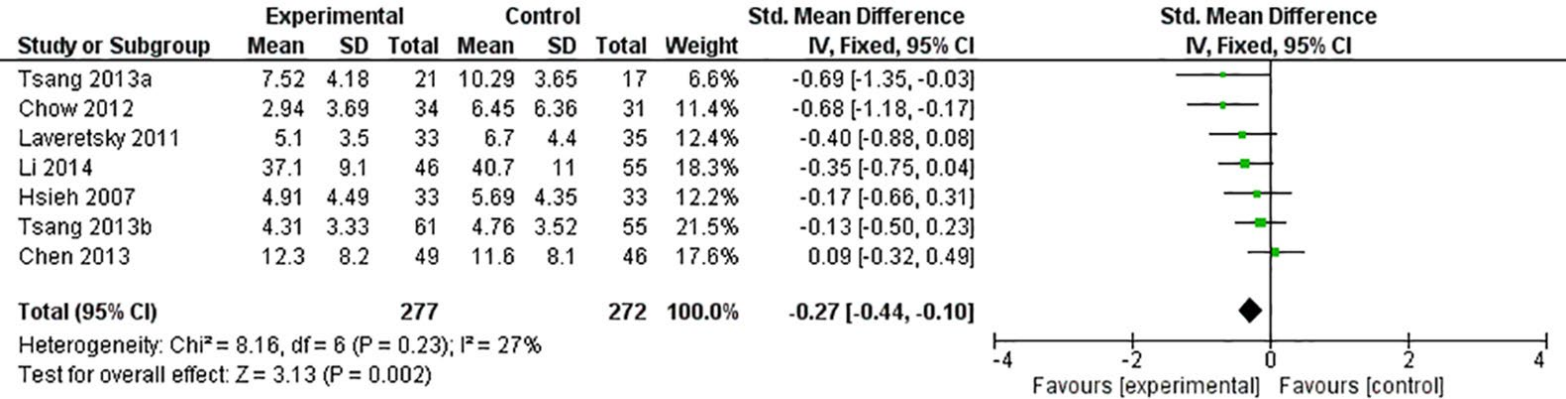
Forest plot of depression.

Among the nine included studies, depression was found to have improved in five studies, while no change was observed in the remaining four studies. Two studies were excluded in the meta-analysis due to insufficient information on the scores of depression. Meta-analysis was performed to detect the effect of qigong on depression and a small to medium significant effect was found (SMD = −0.27, *p* < 0.05, *I* ^2^ = 27%; **Figure 2**). Self-reported scales including the Geriatric Depression Scale, Center for Epidemiologic Studies Depression Scale, Depression Anxiety and Stress Scales, Hamilton Depression Rating Score, and Zung’s Self-Rating Depression Scale were used in the included studies.

#### Possible Mechanisms of Qigong on Depression

##### Neuroendocrine: The Hypothalamic–Pituitary–Adrenal Axis

The HPA axis and the cortisol level were studied in six studies. Five studies collected salivary samples and one study collected urine sample for cortisol analysis. Three studies found significantly lower cortisol levels for the experimental group than the control group during post-assessment (34–36). At the same time, two of the studies showed a significant reduction in depression in the qigong group compared to baseline (35, 36), while Chow et al. (34) showed a decrease in depression, which was, however, statistically insignificant (*p* = 0.053). Chow further showed a significantly lower cortisol levels during follow-up assessment in the experimental group (*p* < 0.001).

**Figure 3:**
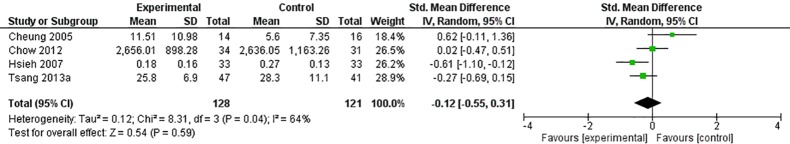
Forest plot of cortisol.

On the other hand, three studies found no significant difference in cortisol between groups across all assessment time points (37, 38, 12). However, two studies found that there was a significant decrease in depression in midpoint and post-assessment, while one study found no change in depression. Meta-analysis was performed on the cortisol levels and no significant effect was found (SMD = −0.12, *p* = 0.59, *I* ^2^ = 64%) (**Figure 3**).

##### Neuroendocrine: Renin–Angiotensin System

Aldosterone and renin were studied in one study (37). Cheung et al. (37) showed no significant difference in aldosterone between two groups. In addition, renin was reported to have decreased significantly for both experimental and control groups. For the symptoms of depression, no significant difference between two groups was observed.

##### Neurotransmitters: Serotonin

The level of serotonin was studied in one study (12). While Tsang et al. (12) found a significant decrease in depression at post-assessment, they found no significant difference in serotonin compared to baseline and no group × time interaction effect was observed. **Table 1** summarizes the results of the neuroendocrine outcomes of the included studies.

**Table 1:** Summary of the neuroendocrine mechanism of qigong.

Author (year)	Study design	Participants	Sample size	Intervention	Control	Depression scales	Outcome measures	Results
Tsang et al. (12)	RCT	Depressed elders with chronic illness	Saliva EG:13/CG:11 Blood EG:14/CG:16	Eight-Section Brocades (12 weeks; 3 times per week; 45 min)	Newspaper reading and discussion program	GDS	1. Salivary cortisol 2. Blood serotonin	1. Across-time change in cortisol was not significant between groups 2. For the blood serotonin level, the group × time interaction effect was not significant
Chow et al. (34)	RCT	Healthy adults	EG:34/CG:31	Chan Mi Gong (8 weeks under instructor’s supervision and 4 weeks practice at home; once a week; 90 min)	Waitlist control	DASS-21	1. Salivary cortisol	1. In weeks 8 and 12, qigong group had lower cortisol levels than control group
Chan et al. (35)	RCT	University students	EG:18/CG:16	Self-Healing Qigong (10 weeks; twice per week; 60 min)	Waitlist control	DASS-21	1. Salivary cortisol	1. Significant reduction in salivary cortisol from week 1 to week 10 in the qigong group, while no statistically significant change in the control group
Chen et al. (38)	RCT	Breast cancer patients	EG:49/CG:46	Guolin New Qigong (5–6 weeks; 5 times per week; 40 min)	Waitlist control	CES-D	1. Salivary cortisol	1. No significant difference between groups for cortisol circadian slope and cortisol awakening responses
Cheung et al. (37)	RCT	Adults with essential hypertension	EG:47/CG:41	Guolin Qigong (16 weeks; twice per week in the first 4 weeks, then was held monthly; 120 min)	Conventional exercise	BDI	1. Urine cortisol 2. Aldosterone 3. Renin	1. No significant difference in cortisol and aldosterone 2. Renin decreased significantly in both groups
Hsieh et al. (36)	Quasi-experimental	Elders	EG:32/CG:30	Laughing Qigong (4 weeks; twice per week; 50–60 min)	Treatment as usual	GDS	1. Salivary cortisol	1. Experimental group showed lower cortisol levels compared to the control. However, no significant changes in cortisol levels for participants in the experimental group, while there was a significant increase in cortisol levels

EG, experimental group; CG, control group; GDS, Geriatric Depression Scale; DASS-21, Depression Anxiety, Stress Scales; CES-D, Center for Epidemiologic Studies Depression Scale; BDI, Beck Depression Inventory.

##### Autonomic Nervous System

Blood pressure was studied in four studies. One study found that both systolic and diastolic blood pressure was significantly lower in the experimental group compared to the control group after qigong intervention (34). Two studies found no significant differences between group or time (39, 31). All four studies showed no significant difference between or within groups for depression.

**Figure 4:**
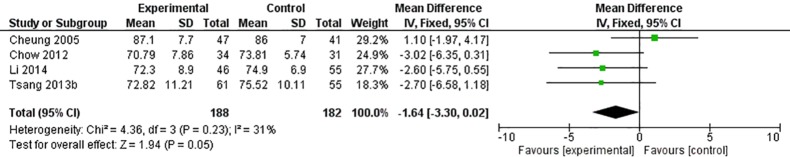
Forest plot of diastolic blood pressure.

Among the two studies which showed significant differences, both studies showed no significant differences in depression (37, 34). Two studies showed a significant difference in depression at post-assessment compared to baseline (40, 41), while three studies showed no significant group and time difference in depression. Only one study showed no significant difference in blood pressure as well as depression across time (39). Meta-analysis was performed on blood pressure, which showed a large and significant effect of qigong interventions on reducing diastolic blood pressure (SMD = −1.64, *p* < 0.05, *I* ^2^ = 31%; **Figure 4**). However, the effect of qigong on systolic blood pressure was not statistically significant (SMD = −0.06, *p* = 0.78, *I* ^2^ = 75%).

Heart rate was studied in three studies and heart rate variability was studied in one study. For heart rate, one out of three studies found a significantly lower heart rate at post-assessment (42), and one study found a significant group × time interaction effect (39). The remaining study found no significant effect of heart rate (34). All of these three studies found no significant difference in depression between groups and times. One study showed no significant change in heart rate and depression (34). For heart rate variability, no significant change was found (31). Also, the same study showed no significant difference between groups in depression. There were enough studies to perform meta-analysis on heart rate. Unfortunately, after Egger’s regression test, studies which involved heart rate showed publication bias (*p* < 0.1). As a result, meta-analysis was not performed on this outcome.

##### Immune System

C-reactive protein (CRP) was studied in two studies (41, 42) and immunoglobin A (IgA) was studied in one study (35). For CRP, Lavretsky et al. (41) found significant between-group difference, while Payne et al. (42) found significant time difference. Lavretsky et al. (41) also showed a significant between-group difference in CRP as well as depression.

Immunoglobulin A was studied in one study (35). Chan et al. (35) showed that the qigong group had a significant increase in IgA at both midpoint assessment (*p* = 0.018) and post-assessment (*p* = 0.018) compared to pre-assessment. While the control group had a trend of increasing in IgA level, it was not significant. The researchers also found a significant reduction in depression in the qigong group compared to baseline. This study provided evidence that qigong was able to demonstrate a significant increase in a mucosal immune marker.

##### Metabolism System

Cholesterol and triglycerides were studied in two studies. One study found only a significant decrease in cholesterol at post-assessment, but not triglycerides. Also, no significance was reported for depression (37). Another study (31) found no significant difference in cholesterol and triglycerides as well as depression.

Blood lipid was studied in one study (31) and found no significant change at post-assessment. There was no significant difference in depression between groups in the same study. Lipoproteins (low- and high-density lipoproteins, LDL and HDL, respectively) were studied in two studies. Both studies found no significant difference between group and time (37, 31). However, Cheung et al. found a significant difference for HDL at post-assessment compared to baseline. Both studies found no significant difference for depression. **Table 2** summarized the results of the physiological outcomes of the included studies.

**Table 2:** Summary of the physiological mechanism of qigong.

Author (year)	Study design	Participants	Sample size	Intervention	Control	Depression scales	Outcome measures	Results
Chow et al. (34)	RCT	Healthy adults	EG:34/CG:31	Chan Mi Gong (8 weeks under instructor’s supervision and 4 weeks practice at home; once a week; 90 min)	Waitlist control	DASS-21	1. Blood pressure 2. Heart rate	1. Qigong group had significantly lower systolic blood pressure and diastolic blood pressure than the control group 2. No group difference on heart rate
Li et al. (31)	RCT	Healthy adults	EG:46/CG:55	Baduanjin (16 weeks; 3 times per week; 30–60 min)	Waitlist control	SDS	1. Blood pressure 2. Heart rate variability (HRV) 3. Rate pressure product 4. Total cholesterol (TC) 5. Triglyceride (TG) 6. Low-density lipoprotein (LDL) 7. High-density lipoprotein (HDL)	1. No significant change was found in blood pressure, HRV, vital capacity, and blood lipid index
Lavretsky et al. (41)	RCT	Elders with major depressive disorder	EG:33/CG:35	Tai chi (10 weeks; once per week; 120 min)	Health education program	HDRS	1. C-reactive protein (CRP)	1. Experimental group had significant decrease in CRP compared to control
Tsang et al. (39)	RCT	Elders	EG:61/CG:55	Yi Jin Ten-Section Brocades (12 weeks; twice per week; 60 min)	Newspaper reading	GDS	1. Hear rate 2. Blood pressure 3. Forced vital capacity 4. Forced expiratory volume	1. Significant group × time interaction effect on resting heart rate 2. For blood pressure, forced vital capacity, and forced expiratory volume, no significant difference was observed
Cheung et al. (37)	RCT	Adults with essential hypertension	EG:47/CG:41	Guolin Qigong (16 weeks; twice per week in the first 4 weeks, then was held monthly; 120 min)	Conventional exercise	BDI	1. Blood pressure 2. Heart rate 3. Total cholesterol 4. Triglycerides 5. LDL 6. HDL	1. Both systolic and diastolic blood pressure decreased significantly in both groups at post-assessment 2. Heart rate was significantly lower for both groups at post-assessment compared to baseline 3. Total cholesterol was decreased significantly in both groups at post-assessment 4. No significant difference was found in triglycerides, LDL, and HDL
Chan et al. (35)	RCT	University students	EG:18/CG:16	Self-Healing Qigong (10 weeks; twice per week; 60 min)	Waitlist control	DASS-21	1.Immunoglobin A	1. Qigong group showed a significant increase in secretion of salivary IgA at post-assessment and follow-up assessment

EG, experimental group; CG, control group; DASS-21, Depression Anxiety, Stress Scales; SDS Zung’s, Self-Rating Depression Scale; HDRS, Hamilton Depression Rating Scale; GDS, Geriatric Depression Scale; BDI, Beck Depression Inventory.

##### Psychological Outcomes

Self-concept and self-efficacy were studied in one study. Tsang et al. (12) studied the change in self-concept before and after the intervention and showed a significant group × time effects of some categories under self-concept. The experimental group experienced lower self-concept in leisure life during midpoint assessment. During the post-assessment, the experimental group showed enhancement of self-concept of physical well-being. For self-efficacy, Tsang found that the experimental group had significantly higher levels of self-efficacy during midpoint assessment (*p* < 0.025). This significant difference was also found in the post-assessment period (*p* < 0.05). At the same time, this study showed a significant decrease in depression for the experimental group in midpoint and post-assessments. Further analysis was done, and it showed that depression was significantly correlated with self-efficacy. Linear regression showed that the change in self-efficacy was able to explain the change in reported depression symptoms. **Table 3** summarizes the results of the psychological outcomes of the included studies.

**Table 3:** Summary of the psychological mechanism of qigong.

Author (year)	Study design	Participants	Sample size	Intervention	Control	Depression scales	Outcome measures	Results
Tsang et al. (12)	RCT	Depressed elders with chronic illness	EC:20/CG:17	Eight-Section Brocades (12 weeks; 3 times per week; 45 min)	Newspaper reading and discussion program	GDS	1.Self-efficacy 2.Self-concept	1.Significant group × time interaction effects on self-efficacy and some categories under self-concept

EG, experimental group; CG, control group; GDS, Geriatric Depression Scale.

### Risk of Bias of the Included Studies

**Figure 5:**
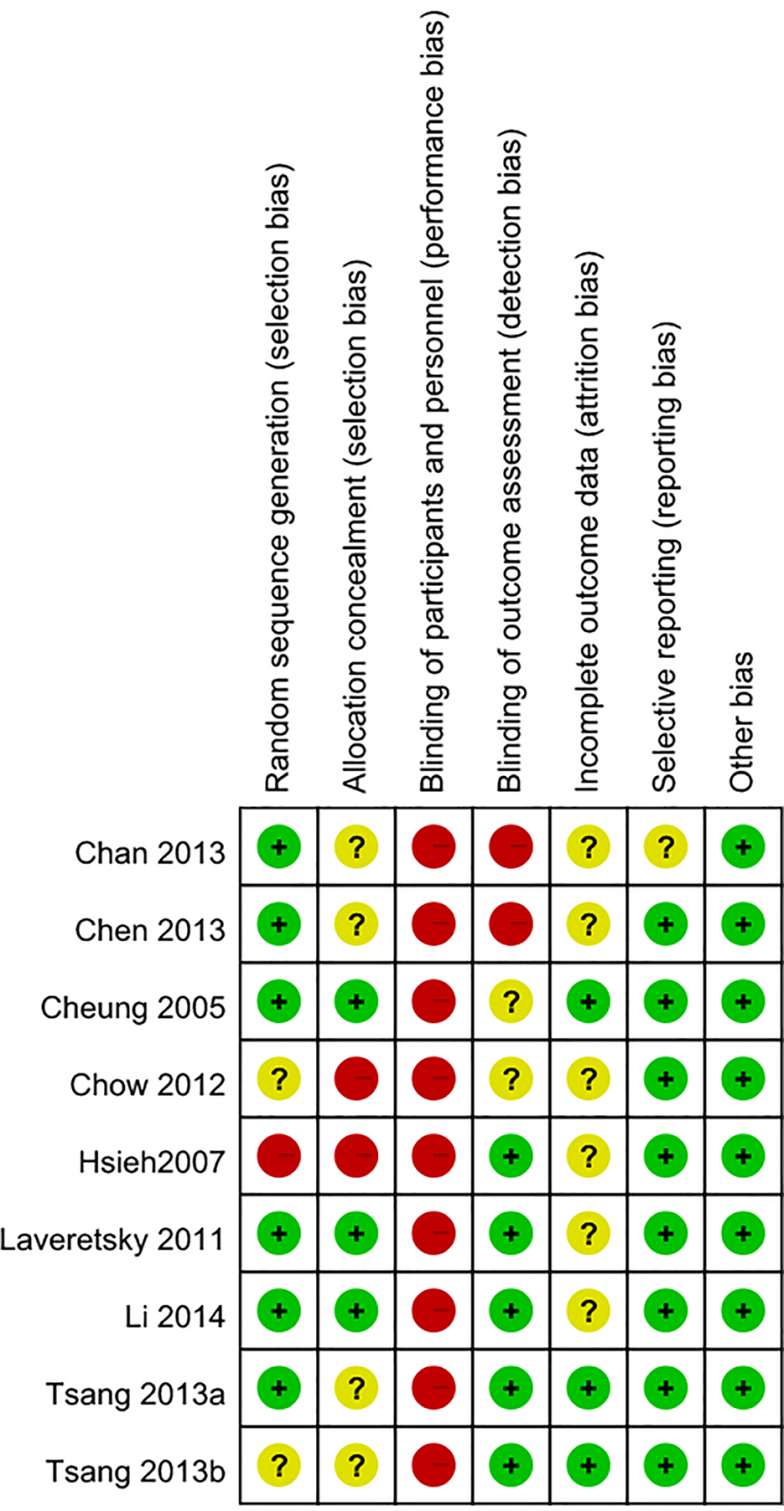
Summary or risk bias.

The risk of bias assessment tools based on Cochrane Reviews were used to assess the quality of the included studies by two reviewers. All included studies had high risk of performance bias (**Figure 5**). Participants were able to identify which groups they belonged to, and this may affect the results of the study. In addition, over half of the included studies were rated as having unknown or high risks in the categories of allocation concealment and incomplete outcome data.

## Discussion

**Figure 6:**
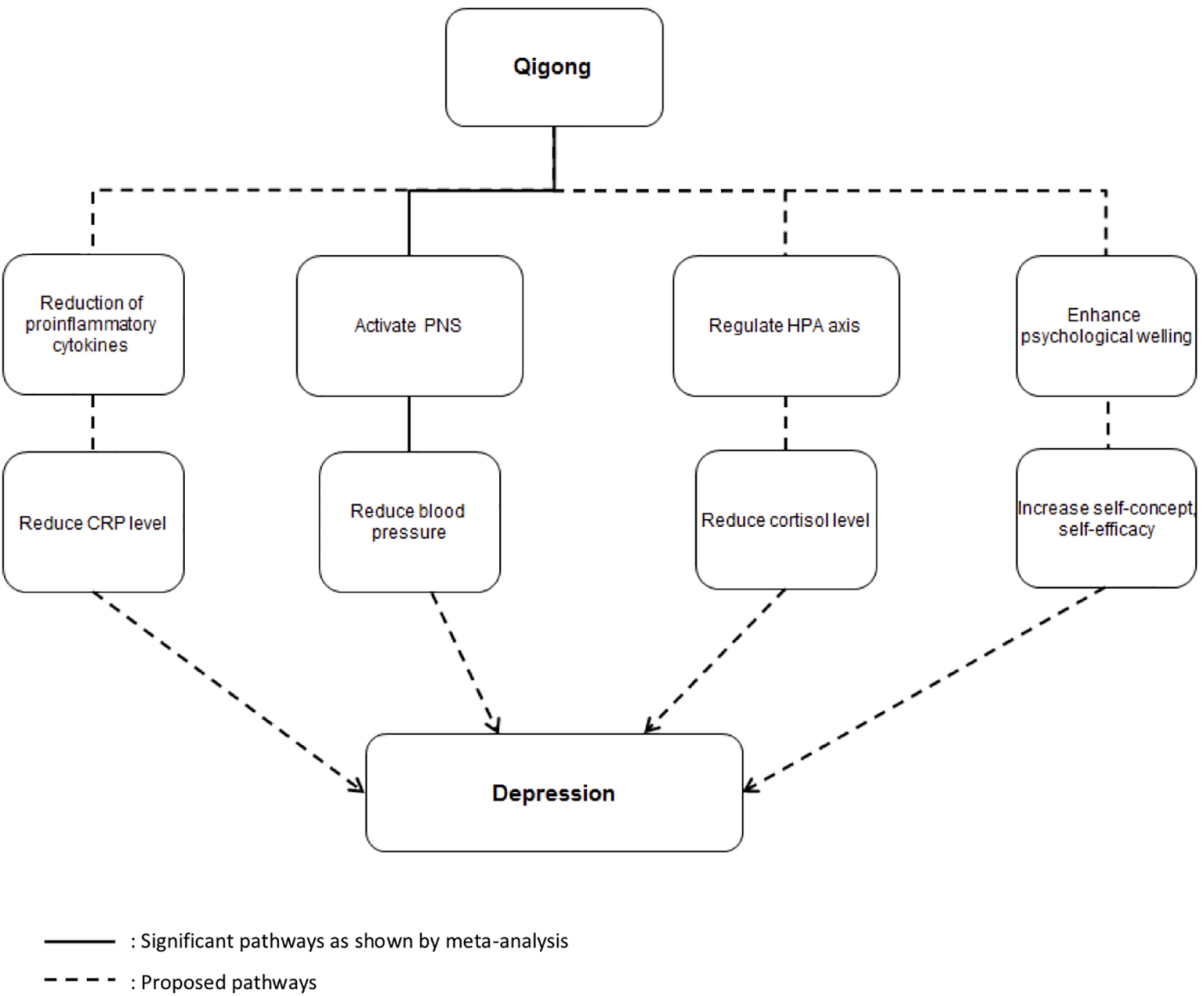
Summary of possible anti-depressive mechanisms of qigong. *Solid lines* indicate significant pathways as shown by meta-analysis. *Broken lines* indicate the proposed pathways.

In this review, participants in the included studies mostly had mild depressive mood. As shown in the meta-analysis, qigong is effective in reducing the symptoms of depression based on the meta-analytical results of seven studies under intensive review. Although the number of weeks and duration of practicing qigong were quite different in the included studies, participants generally showed improvement when they practiced qigong twice a week or more. This is to date the most evidence-based conclusion on the anti-depressive effects of qigong. This review has at the same time further advanced our knowledge on understanding the therapeutic application of qigong by looking into the reasons or mechanisms that may explain this effect of qigong based on existing studies. To summarize the meta-analytical results, the most valid neurophysiological mechanism is that qigong reduces depression by regulating the autonomic nervous system, specifically by upregulating the parasympathetic nervous system. Although our meta-analysis result is based only on blood pressure, which is one of the main indicators of autonomic nervous system (43), it shows a significant effect, as illustrated in **Figure 6**. On the other hand, the effect of qigong on the HPA axis still remains unclear. While meta-analysis shows no significant effect, there is evidence that it can reduce saliva cortisol level (44), and depression is related to the hyperactivity of the HPA axis (45). This meta-analysis cannot provide evidence to confirm the HPA theory, and this may be due to the small amount of available studies.

There are a number of outcomes that did not meet the criteria to be included in the meta-analysis. However, these outcomes may also be indicators of the possible neurophysiological or psychological pathways that can explain how qigong leads to an alleviation in depression. Meta-analysis could not be performed on the outcome of immune system due to the limited number of available studies, and thus the immune system theory could not be supported. Even though some of the outcomes are eligible for meta-analysis, only few studies have investigated the particular outcome. As a result, additional pathways and pathways that need further investigation are proposed and summarized as dotted lines in **Figure 6**. Another possible pathway is that qigong may be able to boost the immune system and thus reduce the level of CRP. This again will lead to a decrease in depression. This is consistent with previous studies showing CRP to be linked to depression (46) and significant reduction of CRP after qigong intervention (47). Last but not least, qigong is essentially a mind–body exercise and it may help to increase psychological well-being by helping individuals develop positive thinking patterns. This may result in an increase in self-concept and self-efficacy and a decrease in perceived stress. The result of this will be a decrease in depression.

There are several limitations with this meta-analytical review. First of all, randomized controlled trials (RCTs) that may be included are limited. RCTs in fact provide the highest level of evidence which is needed in evidence-based practice. If more RCTs are available, the evidence will be more solid and valid as to unraveling the underlying mechanisms of qigong on depression. Second, studies included in this review used different populations with depressive mood. As a result, it is difficult to conclude if the mechanism is related to depression or other comorbid conditions. Also, studies in this line of research used varied types of outcome measures that made meta-analysis difficult. In addition, the types of qigong used were different in different studies. It is hard for researchers to make recommendations on the type of qigong that would be the most beneficial for people with depression. Last but not least, there was no study that examined the neurological mechanism of qigong on depression. Therefore, how our brain perceives and reacts to qigong and how these make changes in the brain remain unknown. Regarding the quality of the existing RCTs, there is much room for improvement. Blinding of participants and personnel may be difficult due to the nature of the intervention. However, other categories such as allocation concealment and incomplete outcome data could be improved by providing more details of the process of the research.

To look ahead, more research efforts should be directed towards exploring the anti-depressive mechanism of qigong. Future studies can use RCTs with higher quality of designs to explore the pathways proposed in this review but have not been confirmed. Also, future studies need to investigate the effectiveness of different types of qigong and its mechanism for people with clinical depression or healthy adults with depressive mood. In addition, future studies can make use of more advanced technologies to look at the neurological mechanism of qigong. For example, the change of brainwaves may be studied by electroencephalogram and event-related potential and the corresponding brain areas that are activated by qigong practice may be studied by fMRI. If researchers are able to put together a more complete picture of the anti-depressive mechanism of qigong, we may have more evidence to inform the clinicians for more effective practice in different clinical settings. It is especially important as qigong is a low-cost treatment which is very easy to get patients engaged in it. Moreover, it does not have any side effects that current medication has (48). Most importantly, if qigong, unlike antidepressants (49), is able to help individuals reduce depression without increasing the risk of hypertension, it could be used as an alternative and/or adjunctive treatment for depression in a safe manner. Based on the results of this study, qigong can be promoted as a preventive intervention so that the public can practice and prevent themselves suffering from clinical depression and other chronic diseases such as hypertension.

## Conclusion

This meta-analytical review has further unraveled the biological underpinnings of the effects of qigong on those with depressive symptoms by providing evidence to bolster its effect to reduce depression through activating the parasympathetic nervous system. Other possible pathways are also proposed, but need to be further tested in future research. Finally, more research is needed to provide solid evidence for the anti-depressive effects of qigong so that clinicians will be able to utilize qigong practice in the clinical settings.

## Data Availability Statement

All datasets analyzed for this study are included in the manuscript and the **supplementary files** .

## Author Contributions

WS wrote the manuscript and conducted data analysis. SC wrote the manuscript. SY and HT wrote the manuscript. All authors reviewed the final manuscript.

## Conflict of Interest

The authors declare that the research was conducted in the absence of any commercial or financial relationships that could be construed as a potential conflict of interest.

## Appendix 1

Search terms and strategies

1. Qigong
2. “Chi gong”
3. “Qi training”
4. EEG
5. FMRI
6. MRI
7. neurofeedback
8. “neurological pathway”
9. neuroanatom*
10. “brain region”
11. neuroimag*
12. “brain structure”
13. Hormone
14. cortisol
15. serotonin
16. dopamine
17. ACTH
18. neurotransmitter
19. “Autonomic Nervous System”
20. “blood pressure”
21. “heart rate”
22. “heart rate variability”
23. “skin temperature”
24. “skin conductance”
25. “galvanic skin response”
26. “Psychological mechanism”
27. “self awareness”
28. mindfulness
29. attitude
30. “emotion regulation”
31. 1 or 2 or 3
32. 4 or 5 or 6 or 7 or 8 or 9 or 10 or 11 or 12
33. 13 or 14 or 15 or 16 or 17 or 18
34. 19 or 20 or 21 or 22 or 23 or 24 or 25
35. 26 or 27 or 28 or 29 or 30
36. (31 and 32) or (31 and 33) or (31 and 34) or (31 and 35)

## References

[1] World Health Organization 350 Million People Suffer From Depression: WHO. Williamsville (2012) USA.

[2] Mathers CD Loncar D Projections of global mortality and burden of disease from 2002 to 2030 (Projections of Global Mortality). PloS Med (2006) 3(11):e442. 10.1371/journal.pmed.0030442 17132052 PMC1664601

[3] Leuchter AF Cook IA Hunter AM Cai C Horvath S Resting-state quantitative electroencephalography reveals increased neurophysiologic connectivity in depression. PloS One (2012) 7(2):e32508. 10.1371/journal.pone.0032508 22384265 PMC3286480

[4] Stahl SM Placebo-controlled comparison of the selective serotonin reuptake inhibitors citalopram and sertraline. Biol Psychiatry (2000) 48(9):894–901. 10.1016/S0006-3223(00)00957-4 11074227

[5] Anacker C Zunszain PA Carvalho LA Pariante CM The glucocorticoid receptor: pivovwt of depression and of antidepressant treatment? Psychoneuroendocrinology (2011) 36(3):415–25. 10.1016/j.psyneuen.2010.03.007 PMC3513407 20399565

[6] Kim Y-K Na K-S Myint A-M Leonard BE The role of pro-inflammatory cytokines in neuroinflammation, neurogenesis and the neuroendocrine system in major depression. Prog Neuropsychopharmacol Biol Psychiatry (2016) 64:277–84. 10.1016/j.pnpbp.2015.06.008 26111720

[7] Leonard BE The immune system, depression and the action of antidepressants. Prog Neuropsychopharmacol Biol Psychiatry (2001) 25(4):767–80. 10.1016/S0278-5846(01)00155-5 11383977

[8] Dowlati Y Herrmann N Swardfager W Liu H Sham L Reim EK A meta-analysis of cytokines in major depression. Biol Psychiatry (2010) 67(5):446–57. 10.1016/j.biopsych.2009.09.033 20015486

[9] Kim Y-K Maes M The role of the cytokine network in psychological stress. Acta Neuropsychiat (2003) 15(3):148–55. 10.1034/j.1601-5215.2003.00026.x 26983358

[10] Tian R Hou G Li D Yuan T A possible change process of inflammatory cytokines in the prolonged chronic stress and its ultimate implications for health. In: Sci. World J. (2014) 2014:8. 10.1155/2014/780616 PMC4065693 24995360

[11] Zunszain PA Hepgul N Pariante CM Inflammation and depression. Curr Top Behav Neurosci (2013) 14:135–51. 10.1007/7854_2012_211 22553073

[12] Tsang HWH Tsang WWN Jones AYM Fung KMT Chan AHL Chan EP Psycho-physical and neurophysiological effects of qigong on depressed elders with chronic illness. Aging Ment Health (2013) 17(3):336–48. 10.1080/13607863.2012.732035 23072658

[13] Henje Blom E Lekander M Ingvar M Åsberg M Mobarrez F Serlachius E Pro-inflammatory cytokines are elevated in adolescent females with emotional disorders not treated with SSRIs. J Affect Disord (2012) 136(3):716–23. 10.1016/j.jad.2011.10.002 22056230

[14] Tsang HWH Fung K A review on neurobiological and psychological mechanisms underlying the anti-depressive effect of qigong exercise. J Health Psy (2008) 13: (7):857–63. 10.1177/1359105308095057 18809635

[15] Yin J Dishman RK The effect of Tai Chi and Qigong practice on depression and anxiety symptoms: a systematic review and meta-regression analysis of randomized controlled trials. Ment Health Phys Activity (2014) 7(3):135–46. 10.1016/j.mhpa.2014.08.001

[16] Koh TC Qigong–Chinese breathing exercise. Am J Chin Med (1982) 10(1–4):86. 6763845 10.1142/S0192415X82000142

[17] Vergeer I Bennie J Charity M Harvey J Biddle S Eime R Participation trends in holistic movement practices: a 10-year comparison of yoga/Pilates and tai chi/qigong use among a national sample of 195,926 Australians. BMC Complementary Altern Med (2017) 17(1):296. 10.1186/s12906-017-1800-6 PMC5461749 28587599

[18] Tsang HWH Cheung L Lak D Qigong as a psychosocial intervention for depressed elderly with chronic physical illnesses. Int J Ger Psy (2002) 17: (12):1146–54. 10.1002/gps.739 12461764

[19] Chow W.-y.Y. The effects of qigong on reducing stress, anxiety and enhancing body-mind wellbeing. Dept. of Applied Social Sciences, The Hong Kong Polytechnic University (2011) Hong Kong.

[20] Guo Y Shi H Yu D Qiu P Health benefits of traditional Chinese sports and physical activity for older adults: a systematic review of evidence. J Sport Health Sci (2016) 5(3):270–80. 10.1016/j.jshs.2016.07.002 PMC6188612 30356513

[21] Xiong X Wang P Li X Zhang Y Qigong for hypertension: a systematic review. Medicine (2015) 94(1):e352. 10.1097/MD.0000000000000352 25569652 PMC4602820

[22] Lauche R Wayne P Dobos G Cramer H Prevalence, patterns, and predictors of T’ai Chi and Qigong use in the United States: results of a nationally representative survey. J Altern Complementary Med (2016) 22(4):336–42. 10.1089/acm.2015.0356 26981616

[23] Ye J Cheung WM Tsang HWH The neuroscience of nonpharmacological traditional chinese therapy (NTCT) for major depressive disorder: a systematic review and meta-analysis. Evidence-Based Complementary Altern Med: eCAM (2019) 2019:2183403–2183403. 10.1155/2019/2183403 PMC6541968 31223326

[24] Zou L Sasaki JE Wang H Xiao Z Fang Q Zhang M A systematic review and meta-analysis of baduanjin qigong for health benefits: randomized controlled trials. Evidence-Based Complementary Altern Med (2017) 2017: 17. 10.1155/2017/4548706 PMC5359459 28367223

[25] Wang C-W Chan CHY Ho RTH Chan JSM Ng S-M Chan CLW Managing stress and anxiety through qigong exercise in healthy adults: a systematic review and meta-analysis of randomized controlled trials. BMC Complementary Altern Med (2014) 14:8–8. 10.1186/1472-6882-14-8 PMC3893407 24400778

[26] Ng BHP Tsang HWH Psychophysiological outcomes of health qigong for chronic conditions: a systematic review. Psychophysiology (2009) 46(2):257–69. 10.1111/j.1469-8986.2008.00763.x 19170945

[27] Klein P Schneider R Rhoads C Qigong in cancer care: a systematic review and construct analysis of effective Qigong therapy. Supportive Care Cancer (2016) 24(7):3209–22. 10.1007/s00520-016-3201-7 27044279

[28] Liu X Clark J Siskind D Williams GM Byrne G Yang JL A systematic review and meta-analysis of the effects of Qigong and Tai Chi for depressive symptoms. Complement Ther Med (2015) 23(4):516–34. 10.1016/j.ctim.2015.05.001 26275645

[29] Tsang HWH Fung K Chan A Lee G Chan F Effect of a qigong exercise programme on elderly with depression. Int J Ger Psy (2006) 21: (9):890–7. 10.1002/gps.1582 16955451

[30] Lee MS Kang C-W Lim H-J Lee M-S Effects of Qi-training on anxiety and plasma concentrations of cortisol, ACTH, and aldosterone: a randomized placebo-controlled pilot study. Stress Health: J Int Soc Invest Stress (2004) 20(5):243–8. 10.1002/smi.1023

[31] Li R Jin L Hong P He ZH Huang CY Zhao JX The effect of baduanjin on promoting the physical fitness and health of adults. Evid Based Complement Alternat Med (2014) 2014:784059. 10.1155/2014/784059 25050127 PMC4094699

[32] Moher D Liberati A Tetzlaff J Altman DG Preferred reporting items for systematic reviews and meta-analyses: the PRISMA statement. Ann Internal Med (2009) 151(4):264. 19622511 10.7326/0003-4819-151-4-200908180-00135

[33] Zhou L Guo Z Xing G Peng H Cai M Chen H Antidepressant effects of repetitive transcranial magnetic stimulation over prefrontal cortex of parkinson’s disease patients with depression: a meta-analysis. Front Psychiatry (2019) 29(9):769. 10.3389/fpsyt.2018.00769 PMC6362497 30761029

[34] Chow YWY Dorcas A Siu AMH The effects of qigong on reducing stress and anxiety and enhancing body–mind well-being. Mindfulness (2012) 3(1):51–9. 10.1007/s12671-011-0080-3

[35] Chan ES Koh D Teo YC Hj Tamin R Lim A Fredericks S Biochemical and psychometric evaluation of Self-Healing Qigong as a stress reduction tool among first year nursing and midwifery students. Complement Ther Clin Pract (2013) 19(4):179–83. 10.1016/j.ctcp.2013.08.001 24199969

[36] Hsieh CJ Chang C Tsai G Wu HF Empirical study of the influence of a Laughing Qigong Program on long-term care residents. Geriatrics Gerontol Int (2015) 15(2):165–73. 10.1111/ggi.12244 24533887

[37] Cheung BM Lo JL Fong DY Chan MY Wong SH Wong VC Randomised controlled trial of qigong in the treatment of mild essential hypertension. J Hum Hypertens (2005) 19(9):697–704. 10.1038/sj.jhh.1001884 15905884

[38] Chen Z Meng Z Milbury K Bei W Zhang Y Thornton B Qigong improves quality of life in women undergoing radiotherapy for breast cancer: results of a randomized controlled trial. Cancer (2013) 119(9):1690–8. 10.1002/cncr.27904 PMC3852682 23355182

[39] Tsang HWH Lee JL Au DW Wong KK Lai KW Developing and testing the effectiveness of a novel health qigong for frail elders in Hong Kong: a preliminary study. Evid Based Complement Alternat Med (2013) 2013:827392. 10.1155/2013/827392 24109493 PMC3784263

[40] Liu X Miller YD Burton NW Brown WJ A preliminary study of the effects of Tai Chi and Qigong medical exercise on indicators of metabolic syndrome, glycaemic control, health-related quality of life, and psychological health in adults with elevated blood glucose. Br J Sports Med (2010) 44(10):704–9. 10.1136/bjsm.2008.051144 18927159

[41] Lavretsky H Alstein LL Olmstead RE Ercoli LM Riparetti-Brown M Cyr N Complementary use of Tai Chi Chih augments escitalopram treatment of geriatric depression: a randomized controlled trial. Am J Geriatric Psychiatry (2011) 19(10):839–50. 10.1097/JGP.0b013e31820ee9ef PMC3136557 21358389

[42] Payne P Fiering S Leiter JC Zava DT Crane-Godreau MA Effectiveness of a novel qigong meditative movement practice for impaired health in flight attendants exposed to second-hand cigarette smoke. Front Hum Neurosci (2017) 11:67. 10.3389/fnhum.2017.00067 28270757 PMC5318411

[43] Erdogan D Gonul E Icli A Yucel H Arslan A Akcay S Effects of normal blood pressure, prehypertension, and hypertension on autonomic nervous system function. Int J Cardiol (2011) 151(1):50–3. 10.1016/j.ijcard.2010.04.079 20472314

[44] Ponzio E Sotte L D’Errico MM Berti S Barbadoro P Prospero E Qi-gong training reduces basal and stress-elicited cortisol secretion in healthy older adults. Eur J Integr Med (2015) 7(3):194–201. 10.1016/j.eujim.2015.01.002

[45] Lamers F Vogelzangs N Merikangas KR de Jonge P Beekman ATF Penninx BWJH Evidence for a differential role of HPA-axis function, inflammation and metabolic syndrome in melancholic versus atypical depression. Mol Psychiatry (2013) 18(6):692–9. 10.1038/mp.2012.144 23089630

[46] Au B Smith KJ Gariépy G Schmitz N C-reactive protein, depressive symptoms, and risk of diabetes: results from the English Longitudinal Study of Ageing (ELSA). J Psychosomatic Res (2014) 77(3):180–6. 10.1016/j.jpsychores.2014.07.012 25128285

[47] Oh B Butow P Mullan B Clarke S Beale P Pavlakis N Effect of medical Qigong on cognitive function, quality of life, and a biomarker of inflammation in cancer patients: a randomized controlled trial. Supportive Care Cancer (2012) 20(6):1235–42. 10.1007/s00520-011-1209-6 21688163

[48] Zou L Yeung A Quan X Hu X Chan J Wang C Mindfulness-based baduanjin exercise for depression and anxiety in people with physical or mental illnesses: a systematic review and meta-analysis. Int J Environ Res Public Health (2018) 15(2):321. 10.3390/ijerph15020321 PMC5858390 29439556

[49] Licht MMC De Geus JCE Seldenrijk PJA Van Hout GH Zitman WJHF Van Dyck WJHR Depression is associated with decreased blood pressure, but antidepressant use increases the risk for hypertension. Hypertension (2009) 53(4):631–8. 10.1161/HYPERTENSIONAHA.108.126698 19237679

